# Immunogenicity and Contraceptive Potential of a Classical Swine Fever Viral Vector Live Vaccine Strain Containing Pig Gonadotropin-Releasing Hormone

**DOI:** 10.3390/vaccines13101048

**Published:** 2025-10-12

**Authors:** Dong-Jun An, Ji-Hee Shin, SeEun Choe, Young-Hyeon Lee, Min-Kyung Jang, Byung-Hyun An, Gyu-Nam Park, Yun-Sang Cho, Kyung-Soo Chang

**Affiliations:** 1 Virus Disease Division, Animal and Plant Quarantine Agency, Gimcheon 39660, Republic of Korea; andj67@korea.kr (D.-J.A.); shinji227@korea.kr (J.-H.S.); ivvi59@korea.kr (S.C.); yhlee916@korea.kr (Y.-H.L.); mkjang0506@korea.kr (M.-K.J.); changep0418@korea.kr (G.-N.P.); choys@korea.kr (Y.-S.C.); 2 Department of Clinical Laboratory Science, Catholic University of Pusan, Busan 46252, Republic of Korea; 3 Department of Virology, College of Veterinary Medicine and Research Institute for Veterinary Science, Seoul National University, Seoul 08826, Republic of Korea; anbh5043@gmail.com

**Keywords:** CSFV, GnRH, Flc-LOM-GnRHx3, oral, pig

## Abstract

Background: Classical swine fever virus (CSFV) is a highly contagious and fatal disease in pigs and wild boars. While hunting and bait vaccination are effective for CSFV eradication, additional strategies are needed to control wild boar populations. This study aimed to develop an oral vaccine, Flc-LOM-GnRHx3, by inserting gonadotropin-releasing hormone (GnRH) epitopes into the Flc-LOM clone. Methods: The Flc-LOM-GnRHx3 strain was rescued from CPK cells and propagated to high titers in MDBK cells. Male boars (20 weeks old) received three doses (10^5.0^ TCID_50_/ml/dose) of Flc-LOM-GnRHx3 either orally or intramuscularly at 2-week intervals. Anti-CSFV E2 antibodies were detected via immunofluorescence and Western blotting. Results: Both vaccination routes induced anti-GnRH antibodies and reduced testosterone levels. Testis size and weight were slightly lower than controls, with seminiferous tubule and spermatid deformities observed in 52.5% of intramuscularly vaccinated pigs and 20.8% of orally vaccinated pigs. Conclusions: Flc-LOM-GnRHx3 demonstrates potential as a dual-function oral vaccine that can eradicate CSFV and impair reproductive capacity in wild boars, offering a novel approach for integrated disease control and population management.

## 1. Introduction

Worldwide, wild boars are a representative invasive pest species that has a negative environmental impact. Wild boars are a reservoir for a number of diseases that affect domestic pigs, and as such, pose a significant risk to commercial pig production, as well as reducing agricultural productivity and causing major ecological damage [1]. Wild boars are very difficult to control because of their large reproductive capacity and their widespread presence in areas that are inhabited or uninhabited by humans. In general, invasive animals such as wild boars can be controlled by lethal or nonlethal methods [1]. Lethal methods include driving, targeting females in the breeding season, expanding overall capture programs, and poisoning [2,3]. A representative nonlethal method is to reduce the size of the population by reducing their reproductive capacity [4,5]. Methods of reproductive control are divided mainly into surgical and non-surgical. Castration is the most common type of surgical sterilization, but it is associated with morbidity and mortality; also, it is often impractical [6,7] and ineffective in wild boars. Therefore, immunocontraceptive vaccines are one of the most promising strategies [8,9]. Among these, the representative immunocontraceptive vaccine is based on gonadotropin-releasing hormone (GnRH), which is designed to counteract the biological action of reproductive hormones [10]. GnRH (also called luteinizing hormone-releasing hormone) is secreted by the hypothalamus [11,12,13]. The main role of GnRH is to regulate the secretion of follicle-stimulating hormone (FSH) and luteinizing hormone (LH) by the pituitary gland [13,14].

In 1998, a commercial vaccine (two doses, given to male pigs) based on gonadotropin-releasing factor (GnRF) was launched in Australia and New Zealand [15], and has since been sold in more than 60 countries [16]. The purpose of this GnRF vaccine is to control male pig odour and overcome the disadvantages of physical castration, which include pain, stress, and reduced growth. The injectable GnRH vaccine, called GonaCon™, is used not only in domestic male pigs, but also in wild animals such as white-tailed deer [17], female cats [18], female wild boars [19,20], and female feral cattle [21]. However, adverse effects of GonaCon™ have been reported, including granulomatous nodules and sterile abscess formation at the injection site and lymph nodes [17,22]. In addition, GonaCon™ and other injectable contraceptive vaccines have limited applicability to wild boars, mainly because wild boars must be captured before they can be injected.

One way to overcome these shortcomings is to develop an oral vaccine using a bait formulation. Classical swine fever virus (CSFV; genus *Pestivirus*; family *Flaviviridae*), which we intend to use as the basis for an oral vaccine, is still prevalent in many pig-producing countries and is the cause of international trade restrictions [23,24]. CSFV carries a single-stranded positive RNA (approximately 12.3 kb) in the form of an open reading frame that encodes four structural proteins (C, E^rns^, E1, and E2) and eight non-structural proteins (N^pro^, p7, NS2, NS3, NS4A, NS4B, NS5A, and NS5B) [25,26]. Previously, the CSFV LOM strain was constructed as an infectious clone, the virus was recovered, and the virus titre and antigenicity were determined [27].

In the present study, we inserted the porcine GnRH epitope between the E1 and E2 genes of the Flc-LOM clone, recovered the virus, and investigated the optimal conditions for production of the Flc-LOM-GnRH strain. In addition, we confirmed production of anti-CSFV and anti-GnRH antibodies after intramuscular (IM) or oral inoculation of the Flc-LOM-GnRH strain into non-castrated fattening male pigs. Finally, we examined the potential ability of the vaccine to protect vaccinated pigs from virus infection, as well as its effects on reproductive efficacy.

## 2. Materials and Methods

### 2.1. Production of Flc-Lom-Gnrh Using a Classical Swine Virus Viral Vector

A Flc-LOM clone was created using LOM, a CSFV vaccine strain, as a backbone [27]. To increase immunogenicity of the GnRH peptide, three copies of GnRH, a Flc-LOM-GnRHx3 clone was created by aligning in tandem into the region between the end of the E1 gene and the start of the E2 gene of LOM (infectious full-length LOM cDNA = Flc-LOM). A pig-GnRH epitope comprises 10 amino acids (pGlu-His-Trp-Ser-Tyr-Gly-Leu-Arg-Pro-Gly-NH2). The pig-GnRHx3 epitopes comprise GnRH epitope (10 aa)-two aa linked (Ser-Gly)-GnRH epitope (10 aa)-GnRH epitope (10 aa)-three aa linked (Gly-Arg-Leu) during synthesis. This was then inserted into the Flc-LOM cDNA.

### 2.2. Rescue of Flc-LOM-Gnrhx3 Virus

The Flc-LOM-GnRHx3 candidate vaccine gene was synthesized as a viral RNA and then transfected into cloned porcine kidney (CPK) cells to rescue the virus. Briefly, CPK cells were cultured in six-well plates and cultured until approximately 80–90% confluent. Next, the cells were cultured for 30 min with Opti-MEM in the absence of foetal bovine serum (FBS) to maintain them under optimal conditions. To generate the viral RNA-Lipofectamine complex, 1–2 μg of viral RNA was diluted in 100 μL of Opti-MEM in one tube, and 2–3 μL of Lipofectamine 3000 was diluted in 100 μL of Opti-MEM in another tube. The contents of the two tubes were then combined and allowed to react at room temperature for 10–15 min. The viral RNA-Lipofectamine complex was then dropped onto CPK cells in FBS-free medium and incubated at 37 °C/5% CO_2_ for 4–6 h, after which the medium was exchanged with fresh medium containing 10% FBS. The cells were then cultivated for a further 24–72 h prior to assessment of cytopathic effects and immunofluorescence assay (IFA). The IFA method first fixes the cells in 70% cold acetone and reacts them with anti-CSFV monoclonal antibody (MEDIAN Diagnostic Co., Cat. no. 9013, Chuncheon, Republic of Korea) for 1 h. After washing with PBS, they are reacted with FITC-conjugated anti-mouse IgG secondary antibody (Thermo Fisher Scientific Co., Cat. A16066, Rockford, IL, USA) at a 1:200 dilution for 1 h, and then the fluorescence is read under a fluorescence microscope. Additionally, Western blot analysis was performed as follows. The supernatant was centrifuged at 12,000× *g* for 10 min, then purified and mixed with 4 × SDS-PAGE loading buffer. Proteins were separated by SDS-PAGE and transferred onto Immobilon^®^-P PVDF (polyvinylidene difluoride) membranes (Merck Millipore Co., Cat. IPVH08100, Darmstadt, Germany). After blocking with 5% skim milk in TBST (Tris-buffered saline with 0.1% Tween^®^ 20 detergent), membranes were incubated overnight at 4 °C with a mouse monoclonal antibody specific to the CSFV E2 protein (MEDIAN Diagnostic Co., Cat. no. 9013, Chuncheon, Republic of Korea). After washing, membranes were incubated with HRP-conjugated goat anti-mouse IgG (Thermo Fisher Scientific, Cat# 31430, 1:5000 dilution) for 1 h at room temperature. Protein bands were visualized using enhanced chemiluminescence (ECL) and detected with a chemiluminescence imaging system.

### 2.3. Growth of the Flc-Lom-Gnrh Virus in Cells

The cell lines used in this study included MDBK (Madin-Darby bovine kidney cells, ATCC CCL-22), PK15 (porcine kidney cells, ATCC CCL-33), ST (swine testicular cells, ATCC CRL-1746), and LLC-PK1 (epithelial-like pig kidney cells, ATCC CL-101), which were purchased from ATCC (Manassas, VA, USA), and CPK (porcine kidney-derived cell line), which was obtained from the Korea Veterinary Culture Collection (KVCC, https://kahis.go.kr:9000/kvcc/ (accessed on 15 September 2025)). To identify the optimal conditions for generating the highest titers of the Flc-LOM-GnRH virus following transfection with viral RNA–lipofectamine complexes, various culture conditions were tested in triplicate. The LOM-GnRH virus was inoculated onto porcine kidney cell lines (CPK, PK-15, and LLC-PK1), a porcine testicular cell line (ST), and a bovine kidney cell line (MDBK) at a multiplicity of infection (MOI) of 0.01, 0.1, or 0.5 (all of these lines are susceptible to CSFV). In addition, the virus growth rate was examined by culturing cells in three different media (MEMα, RPMI1640, and DMEM) supplemented with different concentrations of FBS (0%, 5%, or 10%). Virus growth over 5 days was then investigated using the finally selected cells, MOI, medium, and FBS concentration. Additionally, to investigate for possible mutations in the Flc-LOM-GnRHx3 virus, the virus was subjected to 20 consecutive passages, and the nucleotide sequence of the E1–GnRHx3–E2 region was subsequently analyzed.

### 2.4. Inoculation of Pigs with Flc-Lom-Gnrhx3

The effects of the Flc-LOM-GnRHx3 virus on anti-CSFV E2 antibody formation, anti-GnRH antibody formation, testosterone inhibition, and sperm formation inhibition in non-castrated fattening male pigs were investigated using two routes of inoculation. Three of nine 20-week-old male pigs were assigned to group 1 and administered three oral doses of Flc-LOM-GnRHx3, with a 2-week interval between doses (Table 1). Three pigs assigned to group 2 received three IM injections of Flc-LOM-GnRHx3 virus. Three pigs assigned to group 3 were used as untreated controls. The oral and IM doses of Flc-LOM-GnRHx3 virus were given at 10^5.0^ TCID_50_/mL (Table 1). All pigs in all groups were bled weekly for 7 weeks post-inoculation with Flc-LOM-GnRHx3 virus, and anti-CSFV E2 and anti-GnRH antibody levels were measured. In addition, all animals were necropsied at the end of the 7 weeks, and the testes were subjected to histological analysis. Histopathological analysis of the seminiferous tubules of the testis and epididymal ducts in fattening male pigs inoculated with the CSFV-LOM-GnRH vaccine candidate was performed by a specialized swine pathology institute (Optifarm Co., Osong, Republic of Korea). All pigs (vaccinated and control groups) were observed daily for clinical symptoms (fever, appetite, diarrhea, cough, activity, etc.) during the experimental period.

### 2.5. Anti-Csfv E2 Antibody Elisa and Serum Neutralization Assay

Commercially available serological ELISA kits were used to measure antibody levels in serum. The titers of anti-CSFV E2 antibodies were measured using the VDPro^®^ CSFV AB c-ELISA kit, which is an E2 protein-based ELISA (MEDIAN diagnostic Co., Cat No. ES-CSF-02, Republic of Korea). Test samples with a (%) PC value ≥ 40 were considered positive for CSFV E2-specific antibodies, whereas those with a (%) PC value < 40 were considered negative. A neutralizing peroxidase-linked assay was performed to detect CSFV neutralizing antibodies. Briefly, porcine serum was serially diluted 2-fold in 96-well plates, mixed with the same amount of CSFV (200 TCID_50_/mL), added to CPK cells, and cultured for 3 days. After culture, the cells were stained using a VECTASTAIN^®^ ABC-HRP kit (Vector Laboratories Inc., Cat no. PK-4000, Newark, CA, USA) after reaction with an anti-CSFV monoclonal antibody (MEDIAN Diagnostic Co., Cat no. 9013, Chuncheon, Republic of Korea), Next, ImmPACT DAB Peroxidase (HRP) substrate (Vector Laboratories Inc., Cat no. PK-4100, Newark, CA, USA) was added, 50 μL per well, and the SN antibody titre was calculated as the dilution factor at which no staining was detected. Based on a previous study, the titers of protective SN antibodies against CSFV were set at 4 (log_2_) for 50% potential protection and 5 (log_2_) for 100% potential protection [28].

### 2.6. In-House Elisa to Measure Anti-Gnrh Levels

An in-house ELISA method based on synthesized pig-GnRH antigens (peptides) was used to detect pig anti-GnRH antibodies. Briefly, the GnRH antigen was diluted to a concentration of 0.5–1 μg/mL in coating buffer (0.05 M Carbonate-Bicarbonate buffer, pH 9.6), and 100 μL was dispensed into each well of a 96-well plate. The plates were coated overnight at 4 °C prior to addition of 200 μL of blocking buffer (5% BSA (bovine serum albumin) in PBS-T 0.05% (*v*/*v*) PBS-Tween20 buffer, pH 7.4) for 90 min at room temperature (RT). The plates were washed three times with PBS-T. Pig blood samples were diluted 100-fold, and 100 μL was added to each well. After 2 h at RT, the plates were washed three times with PBS-T, and anti-pig-IgG HRP (horseradish peroxidase; 1:5000; Thermo Fisher Scientific Inc., Waltham, MA, USA) was added for 1 h. Finally, TMB (3,3′,5,5′-tetramethylbenzidine; Thermo Fisher Scientific Inc., Waltham, MA, USA) was added, 50 μL per well, for 10–15 min, followed by 50 μL of stop solution (2 M H_2_SO_4_). Black luminescence at 450 nm was measured using a SMR Sunrise microplate reader (Tecan Group Ltd., Männedorf, Switzerland). Because the optical density (OD) values of the anti-GnRH ELISA differed widely depending on when (week) the blood was collected, separate cut-off values were determined for each sampling week. The cut-off value was defined as the mean OD of the negative control serum plus three standard deviations (mean + 3SD). The intra-assay and inter-assay coefficients of variation (CV) were 5% and 8%, respectively.

### 2.7. Concentrations of Testosterone

Serum testosterone levels were measured using a porcine-specific competitive ELISA kit (Testosterone (Pig) ELISA Kit, Abnova, Taipei, Taiwan; Cat. No. KA2349) according to the manufacturer’s instructions. The principle is that a sample (serum/plasma) containing an unknown amount of testosterone to be analyzed (unlabelled antigen) is added to a standard amount of conjugated testosterone (labelled antigen). The labelled and unlabelled antigens are then allowed to compete for the high-affinity binding sites on the anti-testosterone antibodies coated onto the plate. Whole blood was collected from the jugular vein, allowed to clot at room temperature, and centrifuged at 2000× *g* for 15 min to separate serum. Serum samples were stored at −20 °C and thawed immediately before analysis. Each well of the pre-coated 96-well plate received 50 μL of either standard (0–20 ng/mL), quality control, or test serum sample, followed by 50 μL of HRP-conjugated testosterone. After 1 h incubation at room temperature, wells were washed five times with 1× wash buffer. TMB substrate (100 μL) was added, and the plate was incubated in the dark for 15 min, followed by addition of 50 μL stop solution. Absorbance was measured at 450 nm using a microplate reader. A four-parameter logistic (4PL) standard curve was constructed using the absorbance values of the known standards, and sample concentrations were interpolated from the curve. Final results were expressed in ng/mL. Samples with testosterone levels ≤ 0.3 ng/mL were interpreted as indicating immunocastration, whereas those with testosterone levels > 1.0 ng/mL were interpreted as indicating normal males.

### 2.8. Statistical Analysis

Data are presented as mean ± standard deviation (SD). Group and time effects were analyzed by two-way repeated measures ANOVA in GraphPad Prism (version 8.0, GraphPad Software, San Diego, CA, USA), with Geisser–Greenhouse correction applied when needed. Normality (Shapiro–Wilk) and variance homogeneity (Levene’s test) were confirmed. Supplementary nonparametric tests (Friedman and Kruskal–Wallis) yielded consistent results. Statistical significance was set at *p* < 0.05 (*) and *p* < 0.01 (**).

## 3. Results

### 3.1. Cloning of Flc-LOM-GnRHx3

**Figure 1:**
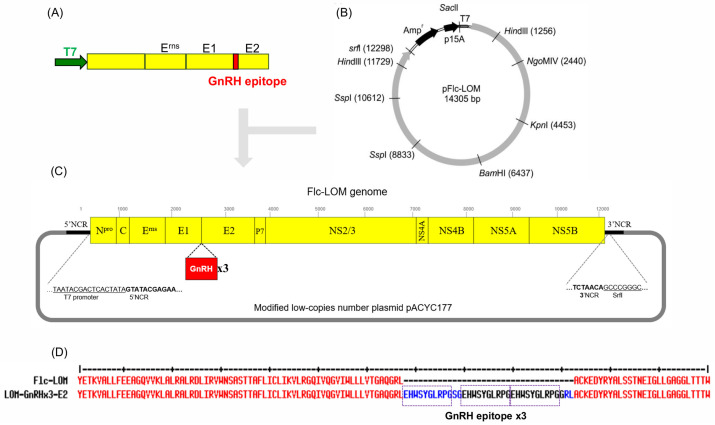
Flc-LOM-GnRHx3 clone generated by insertion of the GnRH epitopes into Flc-LOM. (**A**) The GnRH epitopes were inserted between the E1 and E2 genes of CSFV. (**B**) The pFlc-LOM plasmid containing the Flc-LOM clone. (**C**) Flc-LOM-GnRHx3 was inserted into the modified low-copy-number plasmid pACYC177. (**D**) Comparison of the Flc-LOM-GnRHx3 and Flc-LOM gene sequences.

First, we synthesized a CSFV LOM DNA sequence from the T7 promoter to the end of the E2 gene, with the GnRH epitope sequences inserted between E1 and E2. To insert this synthesized gene sequence into the Flc-LOM clone containing CSFV LOM, the pACYC117 plasmid and clone were treated with *Sac*II and *Ngo*MIV restriction enzymes and then ligated (Figure 1A,B). A modified low-copy-number pACYC117 plasmid containing the GnRHx3 gene was then constructed and named Flc-LOM-GnRHx3 (Figure 1C). The synthesized gene carried repeat GnRH sequences (EHWSYGLRPG-SG-EHWSYGLRPG-EHWSYGLRPG-GRL) between the E1 and E2 genes (Figure 1D).

### 3.2. Rescue of Flc-LOM-GnRHx3 Virus

**Figure 2:**
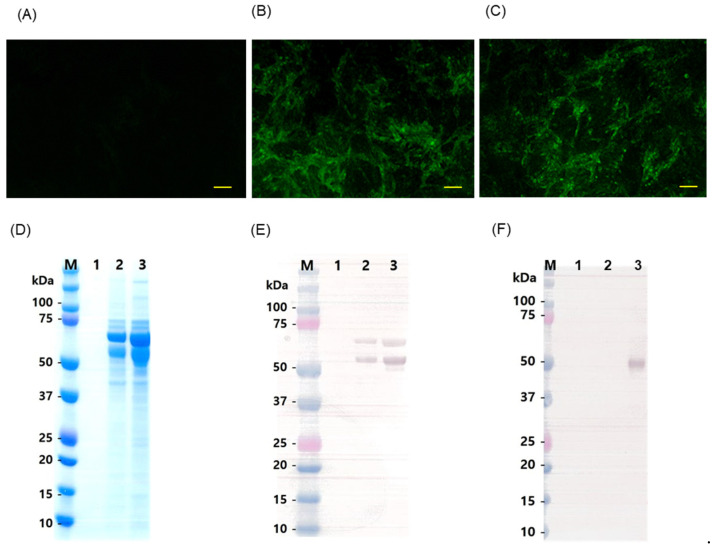
Immunofluorescence analysis (IFA) and Western blotting to detect the Flc-LOM-GnRHx3 and Flc-LOM viruses. IFA with an anti-CSFV E2 mAb was performed to detect (**A**) mock, (**B**) Flc-LOM virus, and (**C**) Flc-LOM-GnRHx3 viruses in CPK cells. (**D**) SDS-PAGE analysis of Flc-LOM virus and Flc-LOM-GnRHx3. Western blotting with an anti-CSFV E2 mAb (**E**) and an anti-GnRH mAb (**F**) antibody detected E2 protein expression (Flc-LOM only) and E2 protein plus GnRH protein expression (Flc-LOM-GnRHx3). Lane 1, mock; lane 2, Flc-LOM; and lane 3, Flc-LOM-GnRHx3. Yellow scale bars = 100 μm.

CPK cells were transfected with Flc-LOM-GnRHx3 linear RNA or Flc-LOM linear RNA (control) using Lipofectamine, harvested after 3 days, and subjected to two more blind passages. CPK cells inoculated at passage 3 were tested by IFA using an anti-CSF E2 monoclonal antibody (Figure 2A–C). IFA confirmed the presence of living viruses in cells inoculated with Flc-LOM-GnRHx3 linear RNA and Flc-LOM linear RNA (as shown by fluorescence emission in the cytoplasm; Figure 2B,C). SDS-PAGE was transferred to Western blot (Figure 2D), and then the expression of Flc-LOM-GnRHx3 and Flc-LOM was confirmed by Western blotting with an anti-E2 monoclonal antibody and an anti-GnRH antibody (Figure 2E,F). Flc-LOM virus reacted with the E2 monoclonal antibody but not with the GnRH antibody (Figure 2E,F); however, the Flc-LOM-GnRHx3 virus was detected by both antibodies (Figure 2E,F).

### 3.3. Optimal Culture Conditions for the Flc-Lom-Gnrhx3 Virus

**Figure 3:**
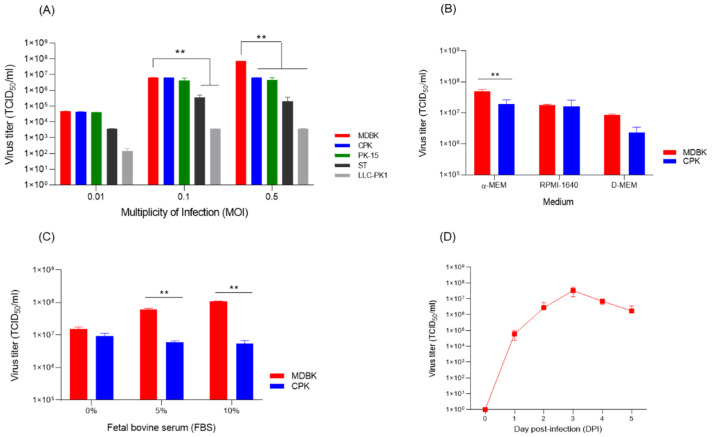
Optimization of cell type, multiplicity of infection, fetal bovine serum content, and harvest time required to maximize Flc-LOM-GnRHx3 virus titres. (**A**) CPK, PK-15, LLC-PK1, ST, and MDBK cells were inoculated with Flc-LOM-GnRHx3 virus at MOIs of 0.05, 0.1, or 0.5. (**B**) Selection of the optimal culture medium (α-MEM, RPMI-1640, or D-MEM). (**C**) Determination of optimal fetal bovine serum concentration (0%, 5%, or 10%). (**D**) Flc-LOM-GnRHx3 virus titres on different days post-inoculation under optimal conditions (cell type, MOI, medium, and % FBS). All culture conditions were tested in triplicate. Error bars represent standard deviations. ** *p* < 0.01.

When the Flc-LOM-GnRHx3 virus was inoculated into five cell types (MDBK, CPK, PK-15, ST, and LLC-PK1) at MOIs of 0.01, 0.1, and 0.5, the highest titre (TCID_50_/mL) was observed in MDBK cells inoculated at 0.5 MOI (Figure 3A). The titre for Flc-LOM-GnRHx3 virus at 0.1 or 0.5 MOI in CPK and PK-15 cells was approximately 10^7.0^ TCID_50_/mL; however, proliferation in ST and LLC-PK1 cells was lower than that in MDBK, CPK, and PK-15 at all MOIs (Figure 3A). Flc-LOM-GnRHx3 virus grew significantly (*p* < 0.01) better in three cells (MDBK, CPK, and PK-15) at an MOI of 0.01 compared to LLC-PK1, and also grew significantly (*p* < 0.01) in two cells (MDBK and CPK) at 0.1 MOI and 0.5 MOI. Proliferation of Flc-LOM-GnRHx3 was highest in MDBK and CPK cells cultured in a-MEM (Figure 3B), and medium containing 10% FBS resulted in higher proliferation than medium containing 5% or 0% FBS (Figure 3C). Regarding culture time, proliferation of Flc-LOM-GnRHx3 in MDBK cells was highest at 3–4 days post-inoculation (Figure 3D). Analysis of the nucleotide sequence of the E1–GnRHx3–E2 region after 20 consecutive passages under Flc-LOM-GnRHx3 virus propagation conditions revealed no genetic mutations, indicating that the virus remained genetically stable during serial cell culture.

### 3.4. Anti-CSFV Antibody Production in Pigs

**Figure 4:**
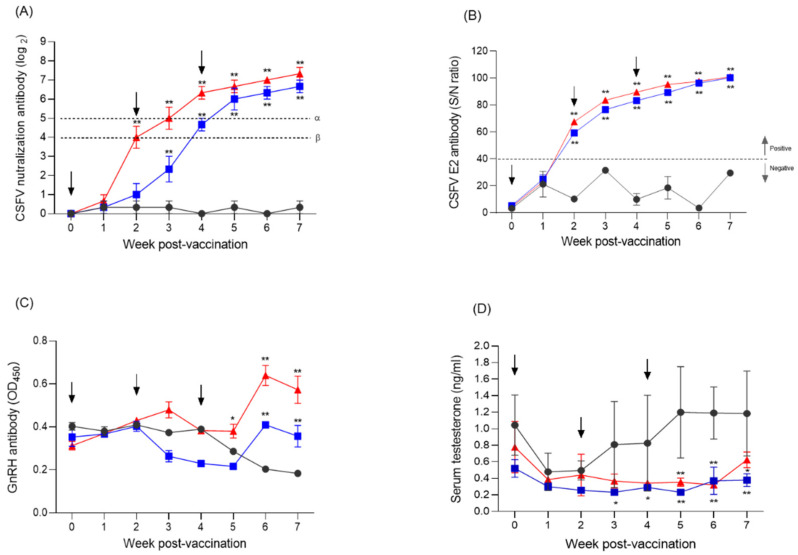
Anti-CSFV serum neutralizing antibody, anti-CSFV E2 antibody, anti-GnRH antibody, and testosterone concentrations in fattening male pigs inoculated orally or intramuscularly with Flc-LOM-GnRHx3 virus. Anti-CSFV serum neutralizing antibody (**A**), anti-CSFV E2 antibody (**B**), anti-GnRH antibody (**C**), and testosterone concentrations (**D**) according to week post-inoculation. Fattening male pigs inoculated orally or intramuscularly with Flc-LOM-GnRHx3 virus are indicated by the blue and red lines, respectively, whereas negative control fattening male pigs are indicated by the black line. In addition, α and β in the CSFV serum neutralizing assay denote 100% potential PA (protection antibody) and 50% potential PA, respectively. Error bars represent standard deviations. * *p* < 0.05; ** *p* < 0.01.

Pigs inoculated IM with Flc-LOM-GnRHx3 virus that generated neutralizing antibodies with a titer of 4.00 (95% CI: 1.52–6.48) (log_2_) provided 50% potential protection at 2 weeks post-vaccination (WPV) while those that generated a titer of 5.00 (95% CI: 2.52–7.48) (log_2_) at 3 WPV provided 100% at 3 WPV (Figure 4A). By contrast, oral inoculation of pigs with Flc-LOM-GnRHx3 virus provided 50% potential protection (4.67 (95% CI: 3.23–6.11) (log_2_)) at 4 WPV and 100% potential protection (6.00 (95% CI: 3.52–8.48) (log_2_)) at 5 WPV (Figure 4A). In addition, neutralizing antibody levels of approximately >7.33 (95% CI: 5.90–8.76) (log2) and >6.67 (95% CI: 5.24–8.10) (log2) were observed at 7 WPV in pigs inoculated IM and orally, respectively, with Flc-LOM-GnRHx3 virus (Figure 4A). The VDPro^®^ CSFV AB c-ELISA detected anti-CSFV E2 antibodies in all pigs in the IM and oral groups from 2 WPV (Figure 4B).

### 3.5. Detection of Anti-Gnrh Antibody and Testosterone Concentrations in Pigs

In pigs inoculated IM with Flc-LOM-GnRHx3 virus, differences in GnRH antibody levels between the control group were first detected 5 WPV, and lasted until 7 WPV (Figure 4C). In addition, differences in GnRH antibody levels between the control group and pigs inoculated orally with Flc-LOM-GnRHx3 virus were first detected at 6 WPV, and lasted until 7 WPV (Figure 4C). By contrast, testosterone levels in pigs inoculated IM or orally with Flc-LOM-GnRHx3 virus began to differ from the control group from 3 weeks (i.e., 3–7 WPV; Figure 4D). In pigs vaccinated with Flc-LOM-GnRHx3, the formation of GnRH is inhibited by anti-GnRH antibodies, and the concentrations of testosterone are also decreased. Testosterone levels must be maintained at ≤0.3 ng/mL to achieve complete contraception. In our experiments, although the vaccinated groups (oral and intramuscular) differed significantly from the negative control, their testosterone levels were slightly above 0.3 ng/mL, indicating that the vaccine may have potential for temporary or incomplete contraception. In addition, no specific clinical signs, such as fever, anorexia, diarrhea, cough, or decreased activity, were observed during the post-vaccination experimental period.

### 3.6. Changes in the Size and Weight of the Testes

**Figure 5:**
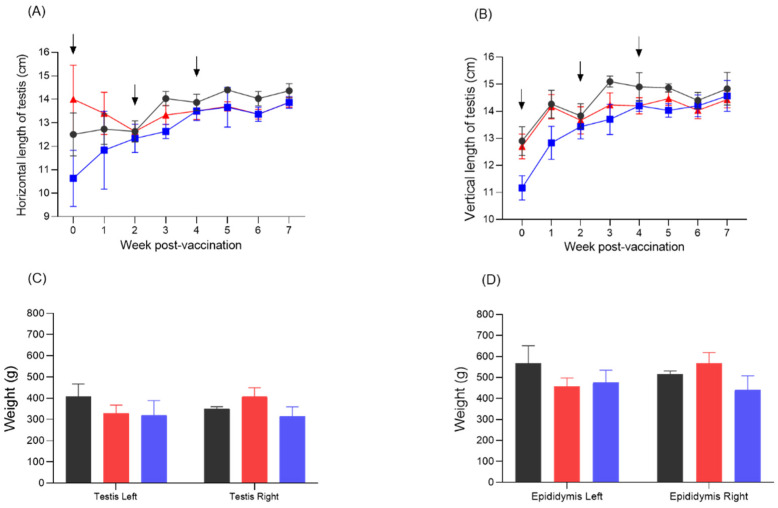
Testicular length, testicular weight, and epididymis weight in fattening male pigs inoculated orally or intramuscularly with Flc-LOM-GnRHx3 virus. Testicular vertical length (**A**) and horizontal length (**B**) according to week post-inoculation. Weight of the left and right testis (**C**) and epididymis (**D**) after autopsy. Fattening male pigs inoculated orally or intramuscularly with Flc-LOM-GnRHx3 virus are indicated by the blue and red lines (or bars), respectively, whereas the negative control fattening male pigs are indicated by the black line or bar. Error bars represent standard deviations.

The horizontal length of the testes of pigs inoculated IM or orally with the Flc-LOM-GnRHx3 virus was similar to that of the control pigs at 2 WPV (Figure 5A); however, differences began to appear from 3 WPV, and the length of the testes in both inoculate groups remained slightly shorter than that in the control group until 7 WPV (Figure 5A). There was also a difference in the vertical length of the testes at 3–5 WPV, but it disappeared almost completely by 6–7 WPV (Figure 5B). The weight of the left testis was slightly lighter in the IM and oral vaccination groups, but the weight of the right testis was the lowest in the oral vaccination group (Figure 5C). This phenomenon was also observed for the left and right epididymis (Figure 5D).

### 3.7. Changes in the Seminiferous Tubules of the Testis, and in the Epididymal Ducts

**Figure 6:**
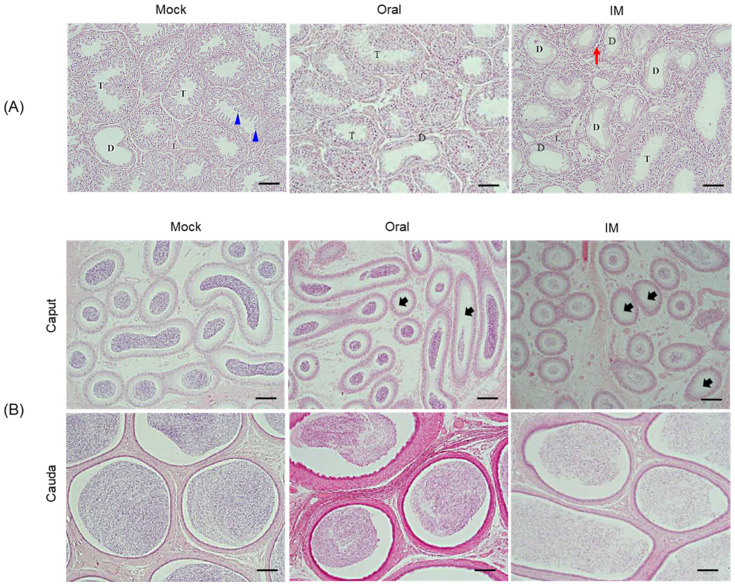
Histopathologic lesions in the seminiferous tubules of the testis and epididymal ducts of fattening male pigs inoculated orally or intramuscularly with Flc-LOM-GnRHx3 virus. Histopathologic lesions in the seminiferous tubules of the testis (**A**) and epididymal ducts (**B**). T: normal seminiferous tubules; D: degenerative seminiferous tubules; L: Leydig cells. Blue arrow: sperm cells; red arrow: inflammatory cell infiltration; black arrow: low sperm density. Black scale bars = 100 μm.

Histopathological analysis of the testis and epididymis revealed no specific abnormalities in the testicular tissue of the control group, and the seminiferous cells in the seminiferous tubules showed a uniform density, with even distribution of differentiated spermatocytes (Figure 6A and Table 2). By contrast, the testicular and seminiferous tubule tissues of pigs inoculated IM or orally with the Flc-LOM-GnRHx3 virus showed local inflammatory cell infiltration, along with undifferentiated and vacuolated testicular cells (Figure 6A and Table 2). The degree of degeneration of seminiferous tubules within the testis was approximately 52.50 ± 0.15% in pigs inoculated IM, and 20.83 ± 0.16% in pigs inoculated orally (Figure 6A and Table 2). Compared with those in the control group, the epididymal ducts of pigs inoculated IM or orally with Flc-LOM-GnRHx3 virus showed a marked or mild reduction in sperm density, respectively (Figure 6B and Table 2).

## 4. Discussion

During mammalian reproduction, GnRH is released by neurons in the hypothalamus; it then travels along the axon and through the portal vein of the central eminence to the pituitary gland, where it binds to its receptors (GnRH receptors) to regulate synthesis and release of LH and FSH by the pituitary gland [29]. As such, GnRH plays a key role in controlling reproduction and is the most common target of contraceptive vaccines. As an endogenous peptide, GnRH does not normally elicit an immune response in animals [29]; however, when GnRH is conjugated to appropriate T helper epitopes [30], and/or combined with delivery systems or adjuvants, it can elicit hormone-specific antibodies [31,32]. Previous studies show that combining the T helper epitope PADRE (P) and the porcine T helper (PT) epitope with GnRH generates P-GnRH and PT-GnRH, respectively [1], and both showed excellent immunogenicity when used to vaccinate animals [1].

Similarly, the PT of the CSFV non-structural protein NS3 transduces T helper responses in pigs [33,34]. The important role of T helpers was confirmed during development of vaccines against CSFV [33,35,36,37,38,39], porcine reproductive and respiratory syndrome virus (PRRSV) [38,40], African swine fever virus (ASFV) [41], and foot-and-mouth disease virus (FMDV) [38]. Various adjuvants and delivery systems have been used to enhance immune responses to peptide antigens [42,43]; however, many of them are toxic or have poor efficacy, particularly when administered orally.

The immunogenic characteristics of LOM, which was used as the backbone for Flc-LOM-GnRHx3, are excellent, and it has protected against CSFV when used as a live CSFV vaccine strain in Korea for more than 40 years [44,45,46]. The LOM vaccine is an emergency vaccine with the advantage of inducing antibody formation in pigs within 7 days of vaccination, and a single vaccination maintains sufficient antibody titres until slaughter [44,46]. The LOM vaccine strain is a commercial vaccine that provides 100% protection at a neutralizing antibody level of 5 (log_2_), and 50% protection at a level of 4 (log_2_) [28]; however, the disadvantage of this vaccine strain is that it is likely to induce abortions, stillbirths, and the birth of weak piglets when administered to pregnant sows that do not have anti-CSFV antibodies, and temporary anorexia and febrile reactions may occur [44,45,46]. These clinical symptoms do not appear in pregnant sows that have anti-CSFV antibodies, and there are no safety issues [45]. The Flc-LOM clone, which harbours 10 amino acids of GnRH (repeated three times to form an endogenous peptide), was inserted between the E1 and E2 genes to generate GnRHx3-E2. When Flc-LOM-GnRHx3 was injected into non-castrated fattening male pigs, neutralizing antibodies more than 16-fold were observed after 2 weeks, and when administered orally, neutralizing antibodies more than 16-fold were observed after 4 weeks. This is similar to observations in a previous study in which neutralizing antibodies were formed 4–5 weeks after oral vaccination with the Flc-LOM-BE^rns^ strain used as a CSFV bait vaccine [47]. In addition, the pigs injected with Flc-LOM-GnRHx3 showed an average OD value of 0.64 in the GnRH assay at 6 weeks, whereas the pigs orally inoculated with Flc-LOM-GnRHx3 showed an average OD value of 0.42 at 6 weeks (the OD value for the control group was 0.21). The blood testosterone levels in pigs injected with Flc-LOM-GnRHx3 were 0.35 ng/mL, those in pigs orally inoculated with Flc-LOM-GnRHx3 were 0.23 ng/mL, and those in the control group were 1.2 ng/mL, at 5 weeks. This means that Flc-LOM-GnRHx3 oral administration to pigs reduces the concentrations of GnRH and testosterone in the blood due to the formation of anti-GnRH antibodies.

We found that the size of the testes and epididymis of pigs vaccinated with Flc-LOM-GnRHx3 by either injection or oral administration was slightly smaller than that in the control group, and there was also a slight difference in weight. In addition, degeneration of the spermatocytes (multifocal degeneration, localized inflammatory cell infiltration in the interstitium) was slightly more prominent in pigs that received the vaccine via injection than in those vaccinated orally. Finally, the sperm density in the epididymal ducts showed a marked decrease in injected pigs, but only a mild decrease in orally-administered pigs. This suggests that Flc-LOM-GnRHx3 has potential as a contraceptive vaccine that affects reproductive capacity when administered orally.

Gonadotropin-releasing hormone (GnRH)-based commercial vaccines have been widely applied for reproductive control and behavioral modulation in various animal species. GonaCon™ (National Wildlife Research Centre, USA) has demonstrated long-term contraceptive efficacy following a single administration in white-tailed deer, feral horses, donkeys, elk, black-tailed prairie dogs, and domestic cats, with generally greater effectiveness observed in females [18,48,49,50,51,52,53]. Improvac^®^ (Pfizer Animal Health, Sydney, NSW, Australia) and Equity™ (CSL, Melbourne, Victoria, Australia) have been shown to suppress sex hormone levels, semen quality, reproductive organ development, and sexual behaviors in pigs, ewes, Asian elephants, and stallions, with certain protocols achieving prolonged contraceptive effects [54,55,56,57,58,59,60]. Bopriva^®^ (Pfizer Animal Health, Parkville, Australia) induces antibodies against GnRF, effectively suppressing testosterone in pre- and post-pubertal bulls, inhibiting testicular function in boars, and reducing progesterone levels, follicular development, and estrous cyclicity in female dairy cows without affecting estrogen levels [61,62,63,64]. Similarly, Improvest^®^ (Zoetis, Kalamazoo, MI, USA) has been employed to reduce sexual behaviors and improve carcass quality in male pigs, as well as to suppress the estrus cycle and enhance growth performance in female pigs [65,66]. Furthermore, Repro-BLOC™ (Amplicon Vaccine LLC, Pullman, WA, USA) was reported to suppress ovarian steroidogenesis, reduce uterine size, and inhibit vascularization in a 59-year-old Asian elephant [67]. Collectively, these GnRH-based commercial vaccines (i.e., GonaCon™, Improvac^®^, Equity™, Bopriva^®^, Improvest^®^, and Repro-BLOC™) provide a non-surgical and versatile approach for long-term reproductive management, behavioral regulation, and production optimization across both wildlife and domestic species. However, their efficacy has been demonstrated only via intramuscular administration [68,69,70]. Development of an effective oral vaccine is difficult because peptides are generally not recognized as antigens in the gastrointestinal tract, are rapidly degraded by proteases, and the tight epithelial cell junctions and thick mucus prevent their systemic absorption; in addition, resistance to peptide antigens develops quickly [71].

Previous studies showed that oral vaccines delivered as baits generate specific antibody responses against CSFV [33,72,73,74,75,76,77], ASFV [78,79], and rabies viruses [80]. We have been spraying bait vaccines (including Flc-LOM-BE^rns^ strain) to control CSFV in wild boars in Korea since 2020 [47], and no outbreaks of CSFV have been reported in wild boars to date. Therefore, we propose replacing the existing CSFV bait vaccine (Flc-LOM-BE^rns^) with a novel bait vaccine containing Flc-LOM-GnRHx3, with the aim of both eradicating CSFV and controlling wild boar populations through contraception. To achieve this goal, further safety studies—focusing on viral shedding and transmission—in both pigs and pregnant sows are required, together with investigations into the horizontal and vertical protective efficacy of the vaccine against CSFV. When administered orally to wild boars, the vaccine might induce only temporary or partial contraception, which would highlight the need for additional studies on its reproductive effects, safety, and efficacy. Furthermore, investigations are needed to optimize bait formulation and dosage, determine the effective administration schedule, and assess viral stability under field conditions.

## 5. Conclusions

We show here that a live Flc-LOM-GnRHx3 vaccine strain without an adjuvant can induce immune responses to CSFV and GnRH after oral administration to non-castrated fattening male pigs. In addition, we observed a certain level of degeneration (multifocal degeneration, localized inflammatory cell infiltration in the interstitium) in porcine seminiferous tubules and a mild reduction in sperm density in epididymal ducts after oral administration.

## Figures and Tables

**Table 1:** Oral and intramuscular inoculation of LOM-GnRHx3 vaccine candidates into non-castrated fattening male pigs.

Group	No. of Pigs	Pig Age	Vaccine	Dose	Route	Inoculation Time/Interval	Autopsy (WPV *)
G1	3	20 weeks	LOM-GnRHx3	10^5.0^ TCID_50_/mL	Oral	Three times/ 2 weeks	7
G2	3	20 weeks	LOM-GnRHx3	10^5.0^ TCID_50_/mL	IM	Three times/ 2 weeks	7
G3	3	20 weeks	Mock	-	-	-	7

WPV *: weeks post-vaccination.

**Table 2:** Seminiferous tubules of the testis and epididymal ducts of fattening male pigs inoculated with the CSFV-LOM-GnRHx3 vaccine candidate.

Group	Seminiferous Tubules of the Testis			Epididymal Ducts
	Diameter (µm)	Degeneration (%)	Histopathologic Lesions	Sperm Density
G1	219.9 ± 15.1	20.83 ± 0.16	Focal degeneration Focal inflammatory cell infiltration into the interstitium	Mild decrease
G2	194.33 ± 35.7	52.50 ± 0.15	Multifocal degeneration Focal inflammatory cell infiltration into the interstitium	Marked decrease
G3	239.43 ± 15.2	6.94 ± 0.05	Nothing remarkable	Nothing remarkable

## Data Availability

The data presented in this study are available from the corresponding author upon reasonable request.
